# Heat Wave Resilient Systems Architecture for Underwater Data Centers

**DOI:** 10.1038/s41598-022-21293-2

**Published:** 2022-10-13

**Authors:** A. A. Periola, A. A. Alonge, K. A. Ogudo

**Affiliations:** Electrical and Electronic Engineering Technology, University of Johannesburg, Johannesburg, South Africa

**Keywords:** Engineering, Electrical and electronic engineering

## Abstract

The need to design computing platforms with low water footprint and enhanced energy efficiency makes non-terrestrial computing platforms attractive. Large scale computing platforms in non-terrestrial environments are increasingly receiving attention. In this regard, underwater data centers (UDCs) are considered to have operational benefits due to their low cooling cost. Underwater data centers experience challenges due to marine heat waves. The occurrence of marine heat waves limits the amount of ocean water available for UDC cooling. This paper proposes a mechanism to detect marine heat waves, and ensure continued UDC functioning. The proposed mechanism utilizes reservoirs to store water and ensure continued functioning of underwater data center. In addition, the proposed research presents the reservoir as a service (RaaS) for ensuring UDC cooling. Furthermore, the presented research also describes modular form factor approach for UDC development. This is being done with the aim of enhancing UDC adoption and use in capital constrained contexts. The underwater data center operational duration is investigated. Evaluation shows that the proposed solution enhances the operational duration by an average of (5.5–12.3) % and (5.2–11.5) % given that marine heat waves span 10 epochs and 15 epochs during an operational phase, respectively.

## Introduction

Future computing relies significantly on data centers and needs to evolve to meet the demands of having enhanced power efficiency. In addition, data centers are expected to have reduced operational costs and low carbon emissions into the environment. These requirements can be met via underwater data center adoption. The development of underwater data center technology is beginning to receive significant attention by technology companies^1^, national authorities^2^ and startups^3^. In comparison to conventional terrestrial data centers, the use of underwater data centers is appealing due to the reduced cooling costs^4,5^, and reduced latency for coastal network subscribers^4,6^. The use of underwater data centers differs from the existing use of terrestrial data centers. This is due to the change from the terrestrial plane to the sub-marine environment. The change to the marine environment necessitates the design of mechanisms for robust functioning of underwater data centers.

However, cold water may not be available at all times due to marine heat wave occurrence. The ocean also hosts energy constrained sensors in underwater internet of things^7–10^. However, sensors have low power consumption and do not generate significant heat. Therefore, it is important to ensure underwater data center maintain their functionality when a marine heat wave reduces the amount of available cold water. This is not considered in^6^.

Akbari et al.^11^ also note that the mesopelagic zone (suitable zone for hosting underwater data centers) has a rapidly changing temperature. Hence, the effect of varying temperature on underwater data centers in the mesopelagic zone should be considered.

The temperature increase in the ocean becomes intolerable to underwater server farms when the ocean’s warm water temperature is unsuitable for cooling. The use of warm water server cooling is an important concept that is important in this regard. Existing work in^12^ have considered the use of warm water for cooling. However, warm water cooling is unsuitable for underwater data centers^12^ as high exit temperatures pose a threat to marine life.

The consideration of the design of solutions that address the challenge posed by marine heat waves to the functioning of underwater data centers is yet to be addressed. Instead, an implicit assumption in^1–3^ implies that underwater data centers will be placed where marine heat waves do not occur. In addition, the adoption and use of underwater data centers is still at a nascent stage. Hence, it is important to consider how marine heat waves influence the performance of deployed underwater data centers. The occurrence of marine heat waves can reduce the effective operational duration of underwater data centers. Therefore, it is important to design solutions enabling underwater data centers to achieve cooling during the occurrence of marine heat waves. This is because uptime is an important performance metric for data center infrastructure. It is important to address the identified operational challenge associated with marine heat waves for underwater data centers. This is because of the usage of underwater data centers in enabling low latency content access for the use case of a coastal subscriber^13^. In addition, underwater data centers have been found to be useful for scientific experiment applications^14^.

### Contribution

The sub-marine location is recognized to be a suitable location for hosting underwater data centers in this regard. The use of underwater data centers is important in Industry 5.0. However, placing data centers in the maritime environment subjects them to new environment challenges. Hence, underwater data centers require robust mechanisms to support their functionality. The discussion proposes robust underwater data centers that are capable of executing data storage and processing i.e. computing tasks in the ocean. The occurrence of marine heat waves poses challenges to underwater data centers (UDCs) executing computing tasks. A mechanism enabling the continued cooling and functioning of UDCs in the event of the occurrence of marine heat waves is proposed. Marine heat waves have been observed to result in a significant perturbation and disruption of marine ecosystems as seen in^15^.

In this case, UDCs experiencing marine heat waves are located in the mesopelagic zone. Marine heat waves result in unexpected and anomalous increase in ocean temperature. The use of ocean water for cooling during a marine heat wave results in exit water having a high temperature that is dangerous to aquatic life. The proposed mechanism enables the UDC to determine the onset of ocean warming and store water in a reservoir for continued service delivery. In addition, the discussion evaluates how the proposed mechanism improves UDC functioning. This is done by formulating the UDC operational duration. The presented research also evaluates UDC operational duration for different reservoir operational efficiencies. Furthermore, the discussion in the research being presented also presents a case where the cooling of UDCs is addressed in the broad context of the blue economy initiative. In this case, UDCs are recognized to benefit from reservoir as a service (RaaS) offering. Furthermore, it is important that capital constrained maritime organizations (CCMOs) are able to adopt the use of UDCs in future computing. This is beneficial as the use of UDCs by more organizations reduce future carbon emissions.

The research is structured as follows: Section “Background and existing work” focus on existing work. Section “Problem description” describes the problem being addressed. Section “Proposed solution: architecture and associated mechanisms” presents the proposed solution. Section “Accelerated adoption: UDC technology” focuses on the performance model. Section “Performance formulation” presents novel approach intended to enhance UDC adoption in capital constrained contexts. Section “Performance evaluation and benefit” describes the results on performance benefits. Section “Conclusion” concludes the research.

## Background and existing work

This section has five parts. The first part discusses work on underwater communications and computing. The related work in this aspect has been considered to evaluate the existing research in underwater communications and related applications. This review demonstrates that existing underwater communications and related applications necessitate using underwater computing platforms. The second part describes work on the interaction between underwater applications and internet access. This discussion is important as it evaluate the existing research in the aspect of integrating underwater applications with the internet. The third part examines existing work in the use of warm water cooling and evaluates the suitability of this technique for cooling underwater data centers in the marine environment. In this case, the existing work is examined to determine if state of the art of the research has considered using the warm water cooling approach with respect to underwater data centers. The fourth part discusses the marine heat wave as an event that should be considered in the deployment and environment friendly operation of UDCs. The relevance of the presented discussion in this regard is that it shows research consideration for the occurrence and influence of marine heat waves on the operation of underwater computing platforms. The fifth part focuses on the perspective of the proposed solution.

### Underwater communications and applications

This aspect of the literature review focuses on discussion work focusing on underwater applications requiring access to computing platforms and resources.

Ali et al.^16^ note that the increased need to conduct coastal surveillance necessitates the use of underwater wireless communications. The discussion in^10^ provides a review of the different challenges that need to be addressed in realizing underwater communications in the context of internet of underwater things. The discussion in^16^ incorporates fifth generation network technologies such as generalized frequency division multiplexing and filter bank multicarrier in underwater networks. A survey that highlights research efforts to address physical layer challenges in underwater wireless networking is presented in^17^.

Underwater communications is used in internet of underwater things applications for ocean monitoring via underwater wireless sensor networks. An underwater sensor network comprises entities such as acoustic sensors, autonomous underwater vehicles and hinges on advances in mobile technology.

Cai et al.^18^ designs a data collection scheme that utilizes the mobile edge computing model. The scheme reduces the unbalanced energy associated with underwater data collection. The data collection is realized via autonomous underwater vehicle path selection methods. Data aggregation and processing involved in the data collection scheme incorporates mobile edge computing. Mobile edge computing reduces data transfer latency associated with processing the data acquired by deployed edge nodes. In^18^, the autonomous underwater vehicles are the data acquisition entities. However, edge computing is not intended to process large data volumes. The use of large scale computing platforms can address this challenge. However, this has not been explicitly considered.

Gregor et al. in^19^ describes the use of underwater gliders to acquire underwater data. They also recognize that there is the challenge of realizing the higher-order underwater data processing. The data acquired by the underwater glider is sent to a processing center via a satellite link. The use of gliders is beneficial due to their low power consumption and compact design. The focus in^17^ is the design and development of GliderTools, an open source package for high order data processing. The discussion examines the usefulness of the open source GliderTools and does not focus on determining the entity that hosts the proposed GliderTools. In comparison to^14,16,17^, the discussion in^19^ does not focus on the design of the network for realizing underwater data processing.

Huang et al.^20^ present research addressing the challenge of data uploading. The data is acquired by underwater sensor nodes. The underwater sensor nodes have the challenge of power and computing resource limitation. The challenge of ensuring that additional computing resources can be accessed to process the acquired data necessitates transmitting to an onshore base station via satellite. Data transmission between underwater sensor nodes is realized via a hop to hop link. The use of compression is proposed due to resource constraints on underwater sensor nodes. The discussion also presents mechanisms that enable data transfer between underwater sensor nodes for delivery to the onshore computing facility. The use of an offshore computing facility is beneficial because it allows underwater sensors to allocate more energy for data acquisition. However, the use of offshore underwater computing facility similar to the onshore computing facility has not been considered.

Alharbi et al.^21^ recognize the usefulness of autonomous underwater vehicles in accessing big data from underwater nodes. This is achieved by periodic visiting of underwater nodes by autonomous underwater vehicles. The use of the autonomous underwater vehicles (AUVs) is advantageous in comparison to relying on a hop to hop link between the power and compute resource constrained underwater nodes. However, the using AUVs is challenging with regard to scaling. This is due to their limited storage capability. The second challenge is the need for alignment between the transmitting (underwater sensor node) and receiving (autonomous underwater vehicle) entities. The alignment is needed when high data rate optical transmission is used. The proposed solution addresses these challenges and presents pipeline architecture. The pipeline architecture comprises underwater nodes with varying data storage and processing capabilities. It addresses the challenge of limited space aboard the autonomous underwater data center. This is realized by either using computing resources aboard nodes to execute a given function or via data set size reduction before being forwarded to a gateway node. The incorporation of central underwater data center computing platform reduces the challenges arising due to the paucity of computing resources in the AUV. This has not been considered in the presented research.

Lima et al.^22^ address the challenge of ensuring interoperability between underwater sensor networks and the internet. They re-design the internet control message protocol for the underwater environment to accommodate the limitations of underwater sensor nodes i.e. scarcity of computing resources resulting in small buffer size. The discussion recognizes the significant challenge arisen from the need to link underwater computing related activities with the internet.

### Underwater data centers: existing approaches

The discussion in this aspect highlights existing research and approach that have focused on the development of underwater data centers.

Underwater data centers have low cooling costs and can reduce the content access latency^4,5^ for coastal network subscribers^6^. The discussion in^23^ describes the functionality and design of an artificial reef data center. Cutler et al.^23^ point out that the artificial reef data center executes computing workload and supports marine life. The design of underwater data centers also benefits from using multiple methods of providing electricity^23^. In addition, energy sources such as fossil—fuels, solar and nuclear power plants can be used to provide electricity to sub-marine assets. Advance in using energy from different sources^24–28^ is important in providing the energy required for operating UDCs.

Currently, UDC utilization does not consider the effects of ocean warming that increases the ocean’s temperature^29^. The discussion in^23^ has not considered how to ensure UDC functionality while considering ocean warming.

### Warm water cooling: method

Water plays a significant role in data center cooling. In this case, the chiller component in data center executes the function of reducing water temperature. The research being examined in this regard is important as it examines the role of chillers in data centers.

Data centers comprise servers that require cooling to preserve the integrity of the constituent electronics. The chiller plays an important role in ensuring the cooling of water to be used for server cooling in server farms. The use of chillers requires the consumption of electricity to realize water cooling and reduction in server temperature. However, the use of chillers is recognized to increase data center energy consumption. A reduction in chiller operational duration can reduce data center energy consumption and also improve the power usage effectiveness. The use of chillers with exit water having high outlet temperature is noted to reduce capital expenditure and cooling costs by up to 40%^30^.

Servers in server farms and data centers do not have 100% utilization at all times. This makes the operation of chillers at all times expensive. The resulting high cost can be reduced by using warm water cooling. The use of warm water cooling method is an approach that appears attractive due to the non-maximal utilization of servers in server farms. Jiang et al.^7^ recognize the suitability of using warm water cooling due to the observed reduction in cooling cost.

Oltmanns et al.^31^ propose using data center waste heat for district warming. In addition^31^, recognizes that improved waste heat utilization can be obtained by supplying the waste heat into the district heating network instead of a campus based buildings only. The proposed solution reduces electrical energy consumption because the use of hot water cooling reduces the need for compression cooling. In addition, the re-use of waste heat reduces Carbon Dioxide emission.

Zhu et al.^32^ identify that the operation of data center chillers results in high power consumption aboard data centers. The controlled and non-operation of chillers at certain epochs reduces the cooling energy and makes use of warm water for server cooling. A strategy for thermal energy recycling from warm water and use it to generate electricity for operating warm water-cooled data centers is proposed in^32^. The intended capturing is done using thermo-electric generators. The discussion in ^32^ differs from^12^ because the thermal energy from warm water is used to operate other warm water cooled servers instead of providing winter heating. The use of thermos-electric generators implies that the approach proposed in^31^ is suitable for use in high temperature locations.

Sartor et al.^33^ examine different data center models with the aim of presenting an open server specification. The discussion recognizes the benefits of liquid cooling such as improved cooling efficiency, enhanced economizer hours and useful waste heat generation. Technology models such as liquid on chip cooling, liquid on board cooling, warm water cooling, and liquid immersion cooling are recognized in the discussion of Sartor et al.^33^. Liquid immersion technology model is similar to the use of ocean water for cooling of data centers immersed in the ocean environment. This approach has the benefits of not requiring chillers, cooling towers and having a significantly low water footprint.

However, the liquid immersion model being presented in the underwater data center approach is different from that presented in^33^. This is because of the different environments. Liquid immersion technologies such as those presented in^33,34^ are intended to function in the terrestrial environment. In the liquid immersion computing systems that have been considered, the coolant supply is limited being constrained by available capital. An ideal system is one in which the supply of coolant is not limited by capital constraints. This can be realized in a system that has a high amount of water such as the ocean.

### New events in the ocean environment: marine heat waves

The discussion in the previous section recognizes the important role of underwater data centers in future computing. Research in cloud computing systems development recognizes the suitability of non-terrestrial computing platforms such as underwater data centers.

However, the ocean is a dynamic environment than surrounding environment of other liquid immersion based coolants. The effect of a varying temperature in this dynamic environment and its influence on server farm cooling should be considered. In addition, the realization of aquatic bio-diversity is an important ocean concern that should be considered. Warm water with temperature in the range 40 °C–45 °C^12,33^ can be used for cooling servers and server farms constituting data centers. The warm water having temperature values exceeding the maximum in this range i.e. 45 °C is not suitable for water cooling. The discussion in^12^ recognizes that warm water with temperature in the range 40 °C–50 °C can be used for the cooling of servers in data centers. However, the use of warm water for cooling data centers in the case of^12^ has considered the case of data centers with low utilization. In addition, the discussion in^12^ involves the integration of thermoelectric cooling into the servers which requires individual server modification. The use of newly defined servers in the context of underwater data centers can result in increased ownership costs. This increase in costs defeats the purpose of using underwater data centers to realize cost savings. In addition, additional information overhead occurs as the thermoelectric cooling system needs to be aware of data describing central processing unit utilization to determine the epochs and duration of desired operation as seen in^12^. The increased information overhead reduces the amount of user data that can be stored aboard servers. The discussion in^33^ identifies that the data center does not solely rely on warm water cooling. Instead, it also makes use of air cooling. However, the realization of air cooling is challenging in non-terrestrial environment such as that presented by the context of the sub-ocean.

The rate of increase in ocean temperature is found to be depth dependent. Meinen et al.^35^ note that the higher rate of increase in temperature per decade occurs with increasing altitude. The ocean temperature increases at 0.04 °C and 0.02 °C per decade at 1360 m and 4757 m, respectively. Buchholz^36^ also notes that there is an increase in sea-surface temperature. The increment in this case is observed to be up to 0.76 °C above the mean sea surface temperature from the twentieth century. In this case, the increment is for the year 2020.

In addition, the ocean temperature can also increase due to the occurrence of marine heat waves that are unexpected epochs of anomalous temperature increase^15,37,38^. Marine heat waves are unpredictable prolonged extreme events in the ocean. Daramaki et al.^15^ note that the study of marine heat waves is just beginning to receive sufficient attention. However, their occurrence is projected to increase with higher intensity. The increase in temperature at the ocean surface is also expected to significantly increase marine heat wave incidences. It is challenging to predict the extent of temperature increase arising from the occurrence of marine heat waves. Marine heat waves increase the temperature of ocean water. The use of the ocean water at elevated temperature for underwater data center cooling results in outlet water having a higher temperature. The resulting higher temperature of the outlet water poses risk to aquatic bio-diversity due to ocean de-oxygenation^39^. Different aquatic life forms have varying dissolved oxygen requirements as seen in^40^. Ocean temperature values of 30 °C and 40 °C have an oxygen content of around 7 mg/L and 6 mg/L, respectively. The oxygen content of 6 mg/L is unsuitable for Trout as seen in^40^. In the event of the occurrence of marine heat waves, an outlet temperature from the data center (water temperature elevated by data center heat) is un-friendly to marine life because it inhibits continued and sustained ocean oxygenation.

Existing research in^12^ and^33^ shows that warm water cooling can be realized for servers in underwater data centers. However, the case in^12^ requires the use of servers that have onboard thermoelectric cooling systems with the intention of reducing the chiller usage thereby reducing energy usage in the data center. However, the use of warm water cooling in the context of^12^ requires having data centers comprising servers having own thermoelectric cooling systems. The use of such servers has not received significant consideration in the case of underwater data centers. Their use and acquisition is potentially associated with increasing ownership costs. In addition, the use of warm water cooling is not intended to replace the usage of cold water but aimed at reducing the energy consumption of chillers in the data centers. It is also recognized in^12^ that significant operational risks accompany the use of warm water for server cooling. The solution in^12^ presents a solution that reduces and not eliminates the risk of server operational failure associated with warm water cooling. The occurrence of marine heat waves that increases the temperature associated with the cooling water therefore poses an operational risk i.e. server failure which should be addressed.

### Identified challenges and solution perspectives

From the discussion, the use of underwater computing platforms is seen to enhance underwater sensor data processing. In addition, UDCs enable low latency content access for coastal subscribers. This is beneficial for low latency access to underwater sensor data. However, UDC operation requires continuous access to the ocean’s cold water. The occurrence of a temporal increase in ocean temperature increases the temperature of ocean water. This reduces the availability of cold water for cooling. The reduced availability poses an operational risk to UDCs. This drawback should be addressed with a focus to ensure UDC functioning.

An additional challenge that should be addressed is ensuring that server payload in underwater data centers has low power consumption. This is because of the need to reduce high costs associated with providing electricity. The power consumption can be reduced by incorporating low power neuromorphic processors instead of conventional von Neumann processors aboard underwater data center servers. This is because neuromorphic processors have low power consumption in comparison to von Neumann processors^41–43^. Neuromorphic processors enable the emergence of new computing entities and are suitable for other types of applications (besides artificial intelligence)^44,45^.

The discussion in this section has recognized the increasing deployment of underwater applications. These applications require access to computing resources. The use of computing resources placed in the underwater environment is identified to be beneficial for identified underwater applications. In addition, aspects relating to the use of different resources for cooling data centers have also been examined.

However, additional research is required to enable underwater data centers deliver the expected functionality in a robust manner. In this case, robust functionality is required with regard to ensuring underwater data centers can function with regard to the occurrence of marine heat waves.

## Problem description

This section presents the problem under consideration. The considered scenario is one comprising an underwater data center located in an ocean region which is experiencing anomalous temperature increase due to the occurrence of marine heat waves. The underwater data center comprises multiple servers being used by subscribers for applications in communication networks^13^, scientific experiments^14^ alongside data storage and algorithm execution. The underwater data center being considered is also being used to host applications in online gaming. The central processing unit aboard the servers in the underwater data center is maximally utilized. In addition, conventional server units without custom made technologies are being used.

The underwater data center is located in an ocean region that is experiencing the occurrence of marine heat waves. In our consideration, marine heat waves occur in phases i.e. different time instants. The occurrence of marine heat waves results in an unexpected increase in temperature. This increase in temperature results in the limited availability of cold water for UDC cooling. The concerned underwater data centers experience rise in their surrounding temperature.

The formulation considers that there are multiple underwater data centers in the underwater environment. Let $\alpha$ be the set of underwater data centers such that:

1 $$\alpha =\left\{{\alpha }_{1},{\alpha }_{2},{\alpha }_{3},\dots , {\alpha }_{N}\right\}$$

In addition, let ${\theta }_{1}\left({\alpha }_{n},{t}_{y}\right), {\alpha }_{n} \epsilon \alpha , t=\left\{{t}_{1},{t}_{2},\dots , {t}_{Y}\right\}$ be the ocean’s temperature around the ${n}{\text{th}}$ underwater data center ${\alpha }_{n}$ at the ${y}{\text{th}}$ epoch ${t}_{y}, {t}_{y} \epsilon t$. The problem formulation considers that the ocean environment hosting underwater data centers experiences variation in temperature between different pairs of epochs. In our consideration, the epoch spans two time instants that define the duration associated with the event of a temperature increase or decrease. The ocean region is deemed unsuitable for hosting UDCs considering the influence of the time epochs and epoch pairs. The ocean region around the ${n}{\text{th}}$ UDC ${\alpha }_{n}$ is unsuitable for hosting UDCs in the case:

2 $$\sum_{{\mathcalligra{f}}\,\,=1}^{a}{\theta }_{1}\left({\alpha }_{n},{t}_{{\mathcalligra{f}}}\,\,\right) < \sum_{{\mathcalligra{f}}\,\,=a+1}^{b}{\theta }_{1}\left({\alpha }_{n},{t}_{{\mathcalligra{f}}}\,\,\right) <\sum_{{\mathcalligra{f}}\,\,=b+1}^{c}{\theta }_{1}\left({\alpha }_{n},{t}_{{\mathcalligra{f}}}\,\,\right)<\sum_{{\mathcalligra{f}}\,\,=c+1}^{Y}{\theta }_{1}\left({\alpha }_{n},{t}_{{\mathcalligra{f}}}\,\,\right) ,{t}_{{\mathcalligra{f}}}\,\,\epsilon t$$

In (2), each individual term described in the summation under consideration is the sum of the ocean temperatures in the time epochs that have been considered. The summation in this case is deemed sufficient to represent the mean temperature in the concerned ocean region. This is because the cardinality of the considered duration (spanning different epochs) is considered equal. In this case, the upper limits of a, b and c are variables that describe the epochs such that the duration in each considered summation span an equal number of epochs.

Another feasible case in the ocean region is one described by the relations:

3 $$\sum_{ {\mathcalligra{f}}=1}^{a}{\theta }_{1}\left({\alpha }_{n},{t}_{{\mathcalligra{f}}}\,\,\right)< \sum_{{\mathcalligra{f}}\,\,=a+1}^{b}{\theta }_{1}\left({\alpha }_{n},{t}_{{\mathcalligra{f}}}\,\,\right)>\sum_{{\mathcalligra{f}}\,\,=b+1}^{c}{\theta }_{1}\left({\alpha }_{n},{t}_{{\mathcalligra{f}}}\,\,\right)<\sum_{{\mathcalligra{f}}\,\,=c+1}^{d}{\theta }_{1}\left({\alpha }_{n},{t}_{{\mathcalligra{f}}}\,\,\right)<\sum_{{\mathcalligra{f}}\,\,=d+1}^{Y}{\theta }_{1}\left({\alpha }_{n},{t}_{{\mathcalligra{f}}}\,\,\right),{t}_{{\mathcalligra{f}}}\,\, \epsilon t$$

4 $$\sum_{{\mathcalligra{f}}\,\,=c+1}^{d}{\theta }_{1}\left({\alpha }_{n},{t}_{{\mathcalligra{f}}}\,\,\right)> \sum_{{\mathcalligra{f}}\,\,=a+1}^{b}{\theta }_{1}\left({\alpha }_{n},{t}_{{\mathcalligra{f}}}\,\,\right), {t}_{{\mathcalligra{f}}}\,\, \epsilon t$$

5 $$\sum_{{\mathcalligra{f}}\,\,=d+1}^{Y}{\theta }_{1}\left({\alpha }_{n},{t}_{{\mathcalligra{f}}}\,\,\right)> \sum_{{\mathcalligra{f}}\,\,=a+1}^{b}{\theta }_{1}\left({\alpha }_{n},{t}_{{\mathcalligra{f}}}\,\,\right),{t}_{{\mathcalligra{f}}}\,\, \epsilon t )$$

In (3), the temperature variation in the duration described by the epochs ${t}_{c} , {t}_{b+1}$ and ${t}_{a+1} ,{t}_{b}$*,* ${t}_{c} \epsilon t ,{t}_{b+1} \epsilon t,{t}_{a+1} \epsilon t, {t}_{b} \epsilon t$ is an increase in the temperature of the concerned ocean region. This is because of the temperature increase and decrease in the duration described by the epochs ${t}_{b} , {t}_{a+1}$ and ${t}_{a} ,{t}_{1}$*;* and the epochs ${t}_{c} , {t}_{b+1}$ and ${t}_{b} ,{t}_{a+1}$ respectively.

The relations in (3), (4) and (5) describe different cases of the comparison of the mean temperature in the underwater environment. In this case, the considered duration span an equal number of epochs (with each epoch comprising different time instants). The case presented in (3) is one considering that an increase in the ocean region due to the occurrence of marine heat waves can result in the mean ocean temperature during some duration (spanning a given epoch) falls short or exceeds the mean ocean temperature in another duration (spanning a different epoch). In (3), there is a reduction in mean temperature of the ocean region surrounding the underwater data center in the last two duration instances. These duration instances span epochs between ${t}_{{\mathcalligra{f}}\,\,=d+1},{t}_{{\mathcalligra{f}}\,\,=d+1} \epsilon t$ and ${t}_{{\mathcalligra{f}}\,\,=Y}$; and epochs ${t}_{{\mathcalligra{f}}\,\,=c+1},{t}_{{\mathcalligra{f}}\,\,=c+1} \epsilon t$ and ${t}_{{\mathcalligra{f}}\,\,=d} ,{t}_{{\mathcalligra{f}}\,\,=d}\epsilon t$.

The scenario in (4) and (5) provides the relations between the mean ocean temperature observed in the duration spanning the epochs ${t}_{{\mathcalligra{f}}\,\,=a+1},{t}_{{\mathcalligra{f}}\,\,=a+1} \epsilon t , {t}_{{\mathcalligra{f}}\,\,=b},{t}_{{\mathcalligra{f}}\,\,=b} \epsilon t$ and ${t}_{{\mathcalligra{f}}\,\,=c+1},{t}_{{\mathcalligra{f}}\,\,=c+1} \epsilon t , {t}_{{\mathcalligra{f}}\,\,=d},{t}_{{\mathcalligra{f}}\,\,=d} \epsilon t$; and ${t}_{{\mathcalligra{f}}\,\,=d+1},{t}_{{\mathcalligra{f}}\,\,=d+1} \epsilon t , {t}_{{\mathcalligra{f}}\,\,=Y},{t}_{{\mathcalligra{f}}\,\,=Y} \epsilon t$ and ${t}_{{\mathcalligra{f}}\,\,=a+1},{t}_{{\mathcalligra{f}}\,\,=a+1} \epsilon t , {t}_{{\mathcalligra{f}}\,\,=b},{t}_{{\mathcalligra{f}}\,\,=b} \epsilon t,$ respectively.

UDCs delivers expected function during the duration described by epochs ${t}_{c} , {t}_{b+1}$ and ${t}_{a+1} ,{t}_{b}$. The functioning of the UDC is infeasible during the set of epochs ${t}_{d} , {t}_{c+1}$ and ${t}_{c} ,{t}_{b+1}$ *;* ${t}_{d} \epsilon t,{t}_{c} \epsilon t,{t}_{c+1} \epsilon t$ due to the increase in surrounding ocean temperature (arising due to the occurrence of the marine heat wave). The increase in the surrounding ocean temperature results in the non-availability of coolants (cold ocean water) for cooling the underwater data center. This reduces UDC functioning duration and essentially the uptime. Nevertheless, it is important that the UDC is able to function during the duration described by the epochs ${t}_{d} , {t}_{c+1}$ and ${t}_{c} ,{t}_{b+1}$. This challenge is addressed in this paper.

## Proposed solution: architecture and associated mechanisms

The discussion presents and describes the proposed solution. The proposed solution is set in the context of an ocean environment with an increasing temperature. In such an ocean environment, the occurrence of marine heat waves becomes highly feasible. Existing research in^35,46,47^ shows that the occurrence of an ocean environment with this profile is applicable to the context of UDCs in our consideration. The discussion in this aspect has been provided to describe the context of the solution being presented for the research problem in the presented mathematical model described by the relations in (1)–(5). The increasing temperature is due to the occurrence of marine heat waves. In this case, the marine heat waves occur at the epoch of maximal server central processing unit utilization. Furthermore, the maximal server central processing unit utilization spans a duration that is equal to the duration of the occurrence of marine heat waves. Hence, the servers have a significant amount of heat generation capacity. In addition, the use of warm water cooling is not considered in this case to prevent or limit the occurrence of failure of servers in the underwater data center. Furthermore, the computing capability of the underwater data center being considered is in high demand by end user and computing platform subscribers.

The discussion in this section has four aspects. The first presents the payload and computing capabilities. The second discusses the UDC assisted cooling approach. The third aspect discusses the innovative service being proposed in the context of the Industry 5.0 initiative. The fourth aspect describes influence of proposed solution on the uptime.

### Proposed solution: enabling payload and computing capabilities

In^3^, the proposed solution aims to find ocean locations suitable for siting underwater data centers. The discussion here extends^3^ by incorporating additional mechanisms and payload. The proposed solution enables the UDC to detect the onset and occurrence of marine heat wave without the use of costly bathymetric studies and surveys. Instead, the proposed mechanism uses novel payload hosted aboard the underwater data center. The payload acquires the temperature ${\theta }_{1}\left({\alpha }_{n},{t}_{y}\right)$ parameter that is used to evaluate the validity of (3). The validity of (3) implies the occurrence of a marine heat wave.

The acquisition of the parameter ${\theta }_{1}\left({\alpha }_{n},{t}_{y}\right)$ is done for epochs ${t}_{y+1} , {t}_{y+2}, {t}_{y+3} ; {t}_{y+1}\epsilon t , {t}_{y+2} \epsilon t , {t}_{y+3} \epsilon t$ and ${t}_{y+r }, {t}_{y+r} \epsilon t$. The temperature in the region (experiencing marine heat wave) hosting the underwater data center ${\mathrm{\alpha }}_{\mathrm{n}}$ at epochs ${t}_{y+1} , {t}_{y+2}, {t}_{y+3}$ and ${t}_{y+r}$ are ${\theta }_{1}\left({\alpha }_{n},{t}_{y+1}\right), {\theta }_{1}\left({\alpha }_{n},{t}_{y+2}\right), {\theta }_{1}\left({\alpha }_{n},{t}_{y+3}\right)$ and ${\theta }_{1}\left({\alpha }_{n},{t}_{y+r}\right)$, respectively. The values of ${\theta }_{1}\left({\alpha }_{n},{t}_{y+1}\right), {\theta }_{1}\left({\alpha }_{n},{t}_{y+2}\right), {\theta }_{1}\left({\alpha }_{n},{t}_{y+3}\right)$ and ${\theta }_{1}\left({\alpha }_{n},{t}_{y+r}\right)$ are determined by the UDC’s temperature sensors. The mean ocean temperature ${\theta }_{exp}({\alpha }_{n})$ is computed aboard the underwater data center, ${\alpha }_{n}$ and given as:

6 $${\theta }_{exp}\left({\alpha }_{n}\right)= \frac{1}{r}\sum_{i=0}^{r}{\theta }_{1}\left({\alpha }_{n},{t}_{y+i}\right).$$

Given that an ocean region hosts multiple UDCs ${\alpha }_{n} , {\alpha }_{n+1} , {\alpha }_{n+2} ;{\alpha }_{n+1} \epsilon \alpha ; {\alpha }_{n+2} \epsilon \alpha$ and ${\alpha }_{n+p} ; {\alpha }_{n+p} \epsilon \alpha$. The UDCs are connected via sub-marine fiber cables and share data on temperature measured at epochs ${t}_{y+1} , {t}_{y+2}, {t}_{y+3}$ and ${t}_{y+r}$ with each other. In this case, the mean ocean temperature, ${\theta }_{exp}({\alpha }_{n},{\alpha }_{n+1} , {\alpha }_{n+2}$*,* ${\alpha }_{n+p})$ is given as:

7 $${\theta }_{exp}\left({\alpha }_{n},{\alpha }_{n+1} , {\alpha }_{n+2}, {\alpha }_{n+p}\right)=\frac{1}{pr} \sum_{s=0}^{p}\sum_{i=0}^{r}{\theta }_{1}\left({\alpha }_{n+s},{t}_{y+i}\right).$$

The acquisition of underwater data center temperature parameters is done by underwater temperature sensors (UTSs) aboard each underwater data center. The operational temperature computing entity (OTCE) receives information from the UTSs and executes (6) and (7). The OTCE determines if the observed mean temperature (and the associated temperature increase) denotes the occurrence of a marine heat wave. This is done via a detection of anomalies in increase in the ocean temperature for a given duration. The detection of anomalies is done by comparing the mean temperature that is computed in (6) and (7) at one epoch with the computational results arising from another epoch.

### Proposed solution: assisted underwater data center cooling

The proposed solution incorporates mechanisms in UDCs that enable them to detect the onset of marine heat wave activity and also store cold water. The storage of cold water enables UDCs to ensure continued cooling when marine heat waves occur. These capabilities are incorporated in the proposed assisted underwater data center cooling mechanism. The storage of the cold water aboard the UDCs can be realized via two types of reservoirs. These are: (1) UDC attached reservoir, and (2) Reservoir as a service (RaaS).

In the proposed solution, the UDC comprises multiple servers. A UDC is noted to be capable of hosting up to 864 servers as seen in the Microsoft prototyping efforts as seen in^48^. However, there is no limitation on the amount of servers that can be hosted aboard a UDC. Therefore, the UDC can host more servers beyond the 864 that has been considered in^48^. The UDC being considered provides data storage, and computing services to a wide range of subscribers and enterprises such as communication networks, scientific institutions and online gaming service providers. It can be used for applications that are currently being executed by conventional terrestrial data centers with key difference lying only in the use of free ocean water for cooling. The considered UDC is connected to terrestrial gateway points via high speed fiber optic cables and benefit from the existing network of underwater fiber optics. In this case, the optic fiber cable network configuration is influenced by the number of terrestrial gateway stations being connected to the UDC. The terrestrial gateway stations are connected to the internet via wireless or wired networks depending on the network context.

The implementation of the proposed mechanism executes two important tasks. These are: (1) Detecting the onset of marine heat wave activity and (2) Reservoir related communications and activation.

In executing the detection of onset of marine heat wave, the assisted underwater data center cooling mechanism ensures UDC cooling when (3)–(5) holds true. Detecting an increase in the mean temperature in successive epochs implies the onset of marine heat wave activity. The detection is followed by the process of storing cold ocean water aboard UDC reservoirs. The stored cold water cools the UDC when marine heat waves occur.

**Figure 1:**
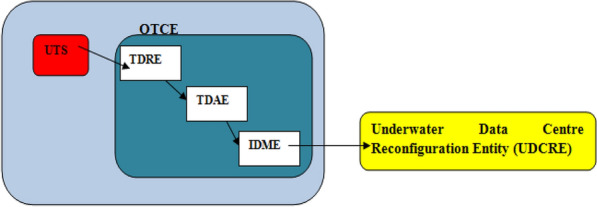
Relation between the UTS, OTCE and UDCRE.

The procedure of reservoir related communications is described in the case of UDC attached reservoir. In this case, reservoir related communications involves the execution of data exchange between the UTS and OTCE entities in the UDC. The relation between the UTS and OTCE in a UDC is shown in Fig. 1. In executing the reservoir related communications, the OTCE incorporates three sub-entities. These sub-entities are the: (1) Temperature data receiver entity (TDRE), (2) Temperature data analysis entity (TDAE) and (3) Inference decision making entity (IDME).

The TDRE receives temperature values from the UTS and sends the received values to the TDAE. The TDAE is the OTCE’s sub-entity that evaluates the validity of (3)–(5). The result of TDAE computation is sent to the IDME. The IDME sends the decision on the non-occurrence or occurrence of temperature increase to the underwater data center reconfiguration entity (UDCRE). The UDCRE transmits signal to the reservoir. If ocean warming is not deemed to begin, the UDCRE continues to receive data from the OTCE.

**Figure 2:**
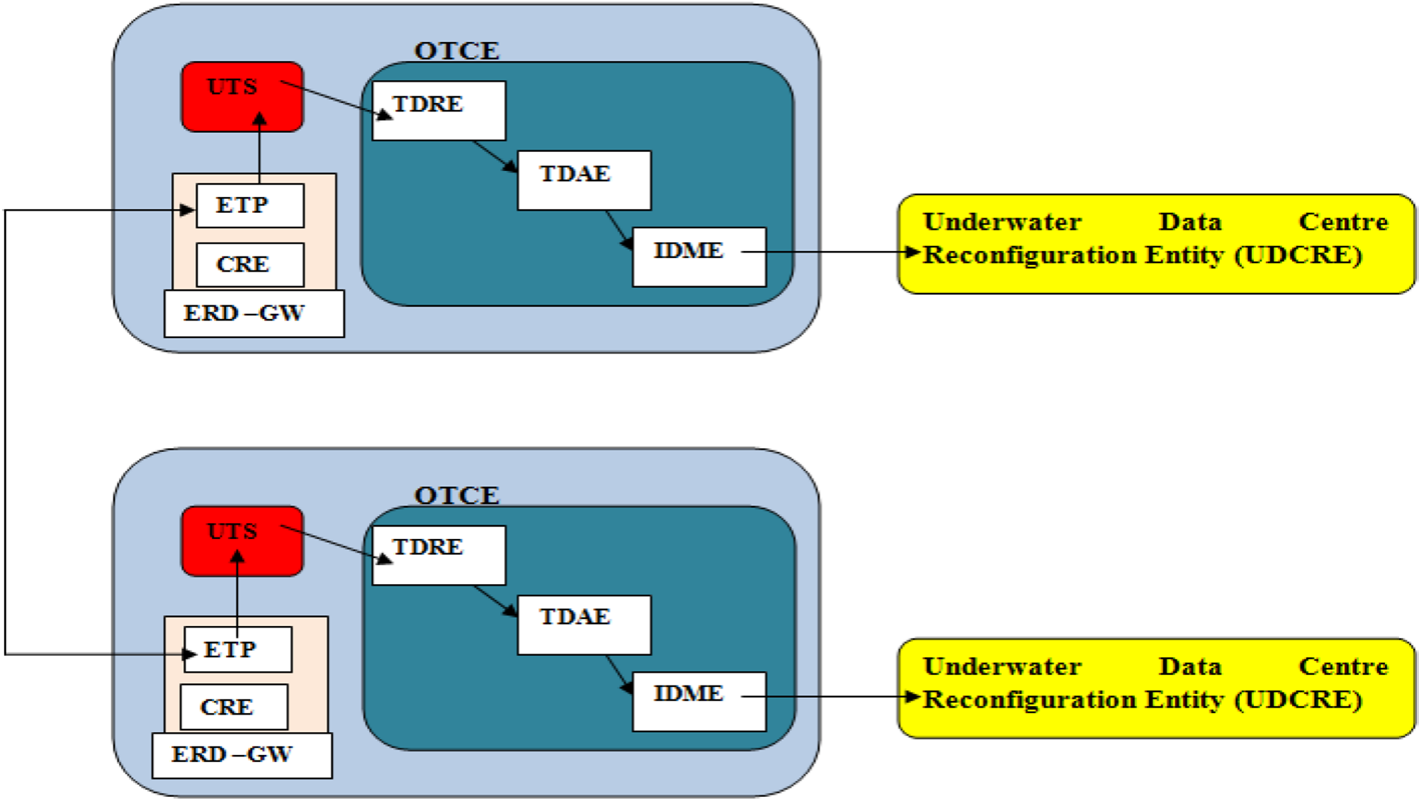
Relations between the ERD–GW and ETP for a case comprising two UDCs.

**Figure 3:**
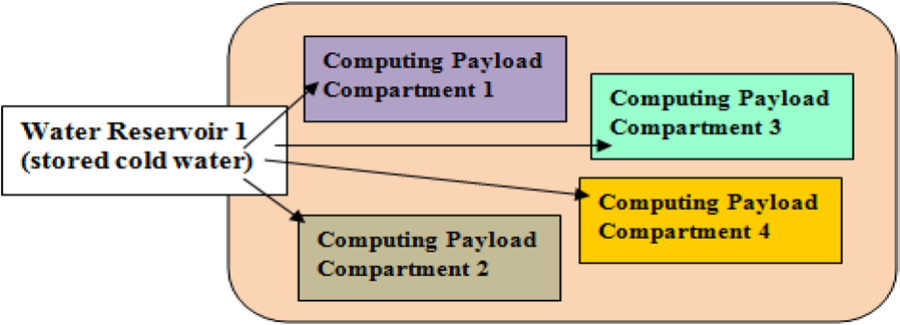
Underwater Data Center with four computing payload compartments and one water reservoir.

**Figure 4:**
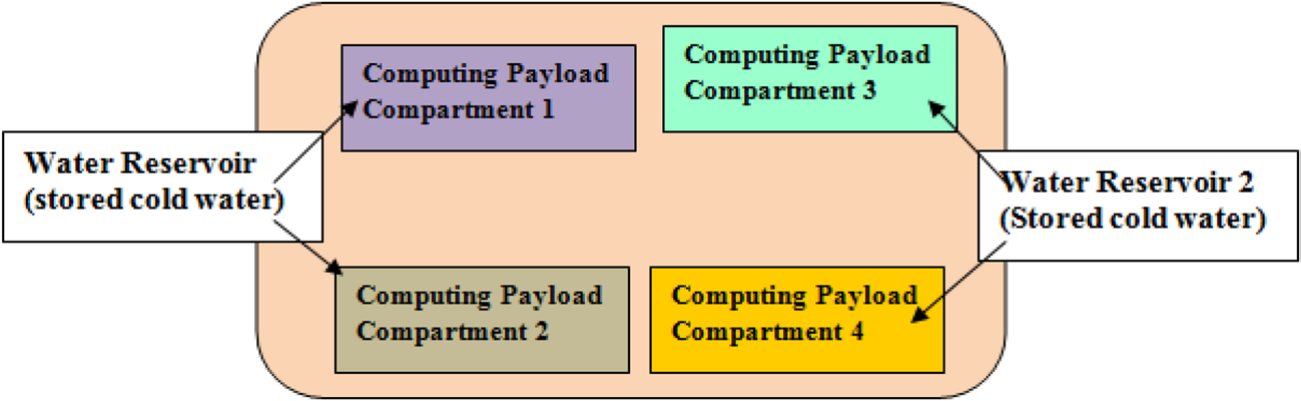
Underwater Data Center with four computing payload compartments and two water reservoirs.

UDC attached reservoirs can also enable functionality in the case of multiple UDCs. In this case, the temperature value acquired by UDCs in different ocean regions is received via the external temperature port (ETP). The ETP is a sub-entity of the external data–gateway (ERD–GW). The ERD–GW hosts the control relation entity (CRE) that receives non-temperature data. Inter ERD–GW communications is realized via sub-marine fiber connections. The relations between ETP, CRE, ERD–GW for two UDCs in the case of UDC attached reservoir is shown in Fig. 2. A UDC (incorporating UDC attached reservoir) can have multiple reservoirs as seen in Figs. 3 and 4. In this case, the concerned UDC incorporates multiple attached reservoirs.

Figures 3 and 4 shows a UDC that has four computing payload compartments with one and two water reservoirs respectively. In Fig. 3, the water stored in the reservoir is shared among the four computing payload compartments. The use of multiple reservoirs is suitable when the computing payload compartment aboard the underwater data center belongs to different computing platform service providers.

In Fig. 3, each reservoir is capable of storing sufficient amount of water capable of cooling maximally utilized servers in the UDC. The dimensions of reservoirs are influenced by the number of servers, server power consumption and the desired server operational duration. In this case, the reservoir can store up to 1.9 million litres of water. This value has been considered because Google is observed to have permissions to pump up to 1.9 million litres of water in^30^. However, the reservoir capacity can be scaled considering the number of servers aboard the UDC system. This is because of the finite number of servers in data center systems. In realizing the proposed cooling, the water stored aboard the reservoir is made available to the computing payload compartment and a heat exchanger executes heat transfer.

### Proposed solution: novel service in industry 5.0

The previous discussion has focused on the design of intelligent mechanism enabling the use of UDC attached reservoir for realizing UDC cooling in the event of marine heat wave. The discussion in this aspect considers the Reservoir as a service (RaaS) as a novel mechanism that is deployed for ensuring UDC cooling and continued functionality. The RaaS enables the delivering of the cooling service for UDCs in the context of the industry 5.0. The incorporation of RaaS provides job opportunities in the blue economy.

The notion of the RaaS considers the context where the deployment of UDCs and ensuring their continued operation considering the occurrence of marine heat waves is decoupled. This decoupling is deemed necessary to consider the context where computing platform service providers seek to avoid the additional overhead arising due to the need to design supporting reservoir systems. In this case, an external entity i.e. a third party develops the system that ensures UDC operational resilience in the event of the occurrence of marine heat waves. This is the framework for the proposed RaaS in the context of industry 5.0. By providing an underwater system component enabling the functioning of UDC (by realizing UDC cooling), the proposed RaaS provides a context for job opportunities in the blue economy from the perspective of technology development and service delivery.

RaaS incorporates ocean based reservoir towers that execute the detection of the onset of marine heat waves and the activation. The reservoir towers comprise multiple reservoir pods. These reservoir pods can be used in conjunction with UDC having own reservoirs or independently to provide cooling for UDCs without integrated reservoirs. Water is stored aboard reservoir towers (having reservoir pods) and delivered to UDCs without integrated reservoirs via water bearing autonomous underwater vehicles (wAUVs).

The proposed wAUVs execute their functionality autonomously. In this regard, the wAUVs are attached to the ocean based reservoir towers and execute the task of communicating with deployed UDCs. The communications enable the wAUVs to obtain information on occurrence of marine heat waves for concerned UDCs. The proposed RaaS incorporates three levels of service providers. These are: (1) Reservoir Tower Provider Entities (RTPEs), (2) wAUV Provider Entities (WPEs), and (3) Robust Communication Service Entities (RCSEs).

RTPEs deploy reservoirs in the sub-marine environment. The deployed reservoirs comprise pods that store and retain cold water in the ocean environment. The reservoir towers host temperature sensors and renewable energy sub-systems that enable the operation of cooling sub-systems for retaining water at very low temperature. Being in a cold environment, low power is required for maintaining the stored water at a cold temperature. Hence, the proposed use of renewable energy is feasible.

WPEs deploy wAUVs that enable the supply of water from reservoir towers to UDCs experiencing marine heat wave events. In providing water to UDCs, wAUVs move water from the reservoir tower to the emptied reservoirs aboard concerned UDCs. RCSEs provide a data transfer service between RTPEs assets i.e. reservoir towers and UDCs. They deploy separate and own AUVs (different from wAUVs) to provide a transfer of data regarding the status of the occurrence of marine heat waves and also affecting the concerned UDC. The AUVs deployed by RCSEs provide information on UDC identifier and the path of sojourn to wAUVs. This enables wAUVs to navigate their path to the concerned UDC.

**Figure 5:**
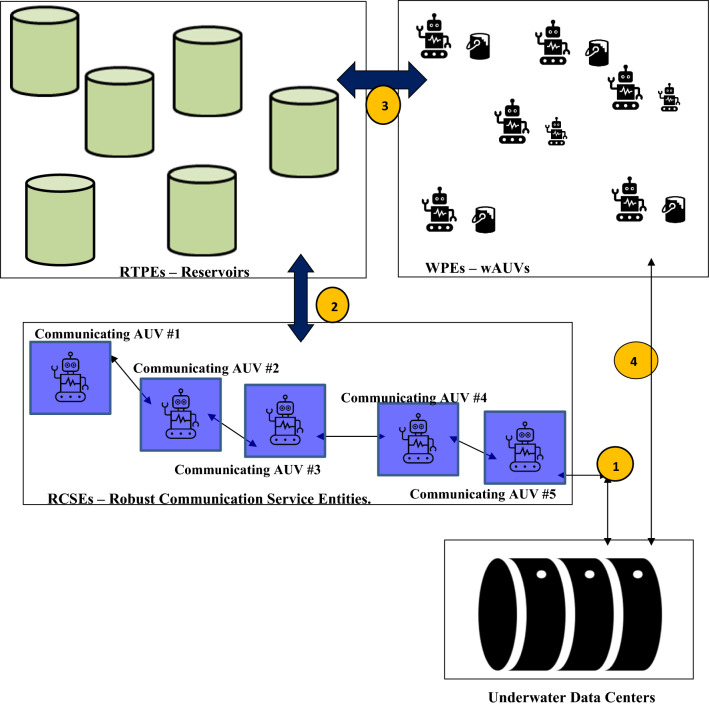
Relations between the RCSEs, RTPEs, WPEs and underwater data centers. (The Figure has been created by AA Periola using Microsoft Word, the logos and objects have been obtained from the Shapes, and Icons options in Microsoft Word).

Relation between the RTPEs, WPEs, RCSEs and an underwater data center (UDC) is shown in Fig. 5. In Fig. 5, RTPEs comprise reservoirs and interact with the RCSEs which provide information on the status of marine heat wave occurrence in the vicinity of a UDC. The relation comprises five communicating AUVs that relay information on the status of occurrence of marine heat wave around a UDC. The relayed information is passed to the RTPE (comprising reservoirs) through the RCSE. In Fig. 5, it is assumed that marine heat waves are occurring. The receipt of this information by the RTPE triggers reservoir supplying water to the wAUVs.

The power usage effectiveness (PUE) is an important metric for data centers an also UDCs. The use of UDCs with benefits of free ocean cooling enables UDCs to have a better PUE performance than existing terrestrial data centers. It is also important to consider the PUE for UDCs given the two types of identified reservoirs. From the perspective of the power usage effectiveness (PUE), the use of the RaaS is more beneficial than the use of UDC integrated reservoirs. This is because the power aboard the UDC is not used to maintain the water at low temperature in the case of the RaaS. However, the power aboard the UDC is used to maintain the water intended for cooling at low temperature in the UDC integrated reservoir.

### Proposed solution: influence on operational duration

The discussion in this aspect presents architecture demonstrating how the incorporation of the reservoir in the underwater data center influences the uptime. The uptime is an important metric that influences operational duration for which the underwater data center can act as a node in communication networks.

**Figure 6:**
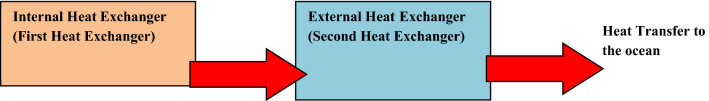
Relations between internal heat exchanger and external heat exchanger in existing scheme.

The incorporation of the water reservoirs influences the UDC power consumption. In the existing UDC; there are two heat exchangers i.e. the internal heat exchanger and the external heat exchanger^1^. The internal heat exchangers are attached to server racks and transfer the resulting heat to water. The hot water is then pumped to heat exchangers which transfer the heat to the surrounding ocean. This two stage heat transfer process does not consider the context where the surrounding ocean experiences a temperature increase. The occurrence of marine heat wave results in a case where transfer of heat (from exiting hot water) to the ocean from the second heat exchanger (external heat exchanger) is infeasible. This arises because of the need to reduce the threats posed by marine heat waves to aquatic life and bio-diversity. The heat transfer process in existing case is shown in Fig. 6.

**Figure 7:**
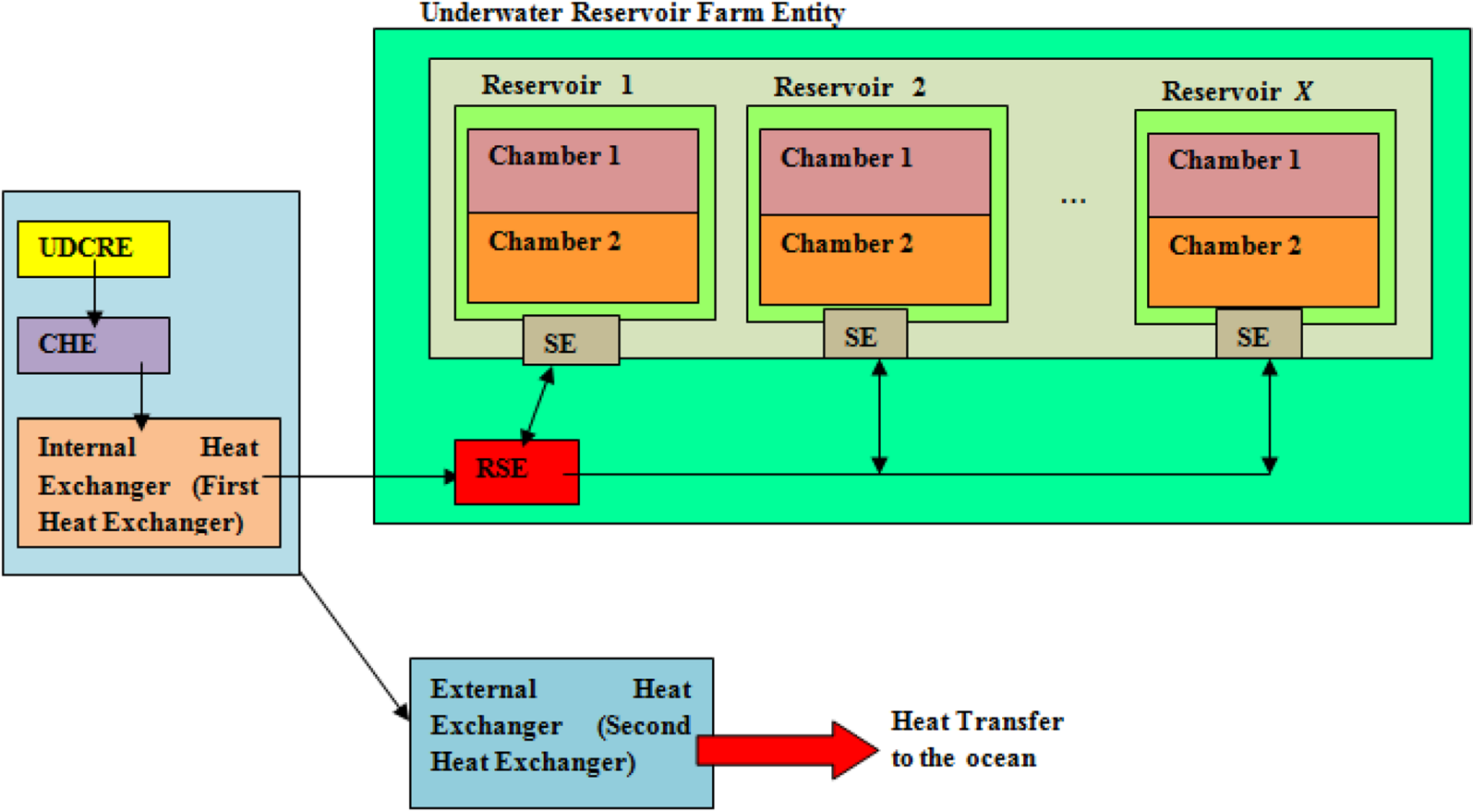
Relations between heat exchangers and reservoir systems in proposed solution.

In the case where the ocean experiences marine heat wave, entities that ensure water supply to the internal heat exchanger for server cooling are required. The reservoirs comprising two chambers execute this functionality. The first chamber stores cold water for supply to the first heat exchanger. The second chamber provides temporary storage for the hot water output from the first heat exchanger. The use of the reservoir is activated when results of decision on marine heat wave occurrence is received from the UDCRE. The relation between the UDCRE and reservoir(s) in the underwater data center is presented in Fig. 7 presenting the role of the cognitive heat exchanger (CHE). The CHE enables the internal heat exchanger to determine if hot water resulting from server cooling should be directed to the external heat exchanger. In the case where marine heat wave event is deemed to occur, the CHE determines the reservoir whose chamber should receive the hot water output. The choice of reservoir is made by the reservoir selection entity (RSE). In Fig. 7, the RSE communicates with the reservoir sensor entity (SE). The SE stores information on the status of each reservoir chamber.

The status indicates if chamber 1 has stored cold water contents (indicating that chamber 2 is empty) or if chamber 1 no longer has stored cold water content (indicating that chamber 2 is not empty). In addition, the SE indicates the utilized chamber capacity of each reservoir. The utilized capacity describes the level to which each chamber hosts the expected chamber content. For example, a chamber 2 with 50% utilized capacity indicates that only half the capacity of the concerned chamber 2 has been utilized. In chamber 1, the utilized capacity indicates the proportion of the stored cold water content that has been used for data center cooling. The underwater data center has multiple reservoirs located in a reservoir farm entity.

In Fig. 7, stored cold water in the first chamber is used for UDC cooling. This is similar to existing system as found in^1^. In the existing system, the pump ensures the transfer of hot water from the internal heat exchanger to the external heat changer and onwards to the surrounding ocean for cooling. In the proposed system, the pump supplies hot water from the internal exchanger to the second chamber of the concerned water reservoir. The pump also uses stored cold water in chamber 1 for data center cooling.

The proposed solution differs from existing case^1^ because it does not require the operation of the external heat exchanger at all operational epochs of the underwater data center. The external heat exchanger does not transfer hot water to the ocean environment during a marine heat wave event. Therefore, the proposed solution reduces energy consumption due to a reduction in the heat exchanger consumed power.

## Accelerated adoption: UDC technology

The discussion in this section presents mechanisms enabling the rapid adoption of UDCs. The considered UDCs comprise both with and without (benefitting from RaaS) integrated reservoirs. The discussion in this section considers the case for the development of UDCs by capital constrained maritime organizations (CCMOs). In this case, CCMOs can benefit from insights observed from the development path of small satellites (as seen in^49^) due to the cost reduction benefit.

A UDC module is designed to be capable of executing computing tasks in a robust manner and comprises two main modules. These are robust service modules (RSMs) and computing executing modules (CEMs). RSMs maintain robust functionality in the event of the occurrence of marine heat waves. The RSMs also host the cooling sub-system that executes modular UDC cooling. CEMs execute the functionality refers to the execution of expected data center functions i.e. receiving, storing and processing data.

UDC modules are similar to small satellites in promoting the goals of ocean colonization via the deployment of multiple UDCs by CCMOs. The proposed modular UDC has the features and capabilities of: (1) Aggregation Capability, and (2) Minimum Size Specification. The modular UDC has a minimum size in terms of physical dimensions and computing capability. This minimum size can be realized considering the CCMO’s constraints. In addition, modular UDC has aggregation capability i.e. modular UDCs can be stacked together to realize a single UDC. Currently, UDCs are deployed and installed as large data center structures and the individual servers or groups of servers can’t be operated in a standalone manner.

A UDC module is designed to be capable of executing computing tasks in a robust manner and comprises two main modules. These are robust service modules (RSMs) and computing executing modules (CEMs). RSMs maintain robust functionality in the event of the occurrence of marine heat waves. The RSMs also host the cooling sub-ystem that executes modular UDC cooling. CEMs execute the functionality refers to the execution of expected data center functions i.e. receiving, storing and processing data.

The use of open source strategies can be used for UDC development by CCMOs. The open compute platform (OCP) as seen in^50–52^ is an open-source strategy that enables enterprise data center service providers to share technology solutions and strategies. However, the OCP focuses only on terrestrial data centers and not UDCs. The increasing adoption of UDCs can be realized by integrating their coverage in the existing OCP framework. This will enable more CCMOs to be capable of accessing technology suitable for UDC development. The UDC adoption framework should incorporate important information elements enabling hardware designers and developers to develop and deploy UDCs.

Information elements should comprise data on the UDC. The required information is in seven categories. These are: (1) Computing Details, (2) Power Access Details, (3) Power Usage Details, (4) Performance Details, (5) Location Information, (6) Vessel Related Information, and (7) Access to information technology.

The information considered in the computing details is: (1) Computing Payload i.e. Number of Servers, (2) Server capacity, (3) Hardware details i.e. manufacturers, specifications and serial number, (4) Required Energy for server operation and (5) Deployed software. The information considered in the power access details is: (1) Accessed renewable energy, (2) Forms of accessed renewable energy, (3) Accessed energy from the grid, (4) Onboard power storage capability, (5) Battery Details i.e. manufacturers, specifications and serial number and (6) Number of batteries. Power usage details consider the electricity consumption associated with: (1) each onboard server, (2) each heat exchanger, and (3) each element in UDC’s support sub-system. Performance details comprises: (1) Observed operational duration, (2) Power usage effectiveness (PUE) and (3) UDC number of failures observed over a pre-determined duration.

The description of maritime resource location is specified in the Location Information aspect as: (1) Type of maritime resource i.e. river, ocean or lake. (2) Name of concerned maritime resource, (3) Location of maritime resource described by surface coordinates, (4) Depth of UDC in the maritime resource and (5) Regulatory permit details. Vessel related information comprises details such as: (1) Class of vessel, (2) Age of vessel, (3) Custom design status and (4) Vessel capacity. The custom design status indicates if the vessel is being re-used or was specifically produced for use as a UDC support vessel.

The UDC is also required to be capable of communicating with external networks and requires access to information systems and networks. The category of ‘Access to information technology’ comprises data on: (1) Accessibility indicator to sub-marine fiber optic cable, (2) Number of accessed fiber optic cables and (3) Link speed.

**Figure 8:**
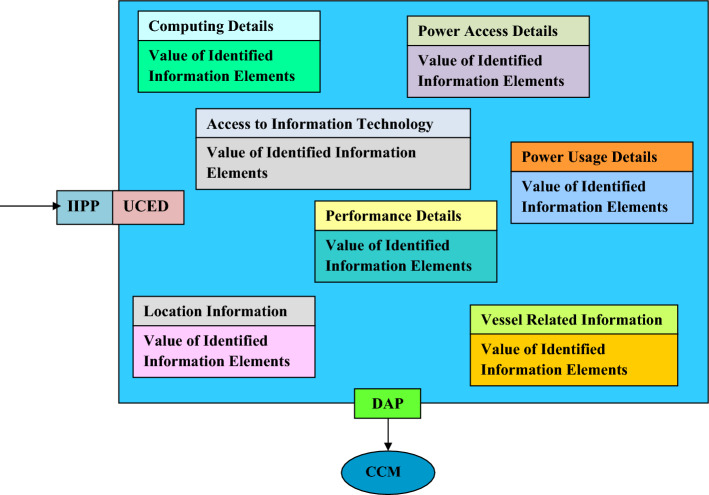
Relations between the IIPP, UCED, DAP, information elements and access by the CCMO.

The information in each category is stored aboard a database by entities deploying UDCs at different global locations. In addition, stored information can be accessed by CCMOs seeking to develop and deploy UDC technology. Service providers that have developed and deployed UDCs upload information in this category into the underwater computing entity database (UCED). The UCED is a sub-component of the OCP. It hosts the information element in each pre-defined and aforementioned category. The upload is done via the information provision portal (IIPP). Prospective hardware designers and service providers that aim to utilize UDCs access uploaded information via the Designer Access Portal (DAP). Relations between the IIPP, UCED, DAP and the information elements is shown in Fig. 8^53^.

## Performance formulation

The performance model is formulated and described in this section. The parameter of interest is the data center uptime. This is influenced by the different phases of operation of the underwater data center. The metric of the power usage effectiveness (PUE) has not been considered. This is because UDCs benefit from free ocean cooling, utilize smaller power for cooling thereby having an improved PUE in comparison to existing terrestrial data centers.

The use of UDCs is still at a nascent stage from the perspective of research and enterprise development. There is still a paucity of system emulation software that can be used to model and examine the performance of the underwater data centers in the context of the presented solution. Hence, it is important to develop mathematical models to formulate the performance model describing the performance of underwater data centers. The role of mathematical models in this case is important to flexibly examine how the sub-ocean environment influences underwater data center performance. In this regard, the formulation of mathematical models serves two purposes. In the first case, the use of mathematical models provides a means for clarifying the parameters that influence the performance of the underwater data center considering the metric of the data center uptime. Secondly, the use of mathematical models provides a means of demonstrating the relations between the parameters that have been identified to influence underwater data center performance.

The performance model is formulated considering the NOPh, ROPh and MWPh. The duration of each of the NOPh, ROPh and MWPh make up functional duration of the underwater datacenter. In the case of the existing scheme, the ROPh and MWPh are not considered. The proposed scheme comprises the ROPh, MWPh and NOPh in our consideration.

The proposed solution introduces the reservoir and associated mechanisms (active in the ROPh) to ensure all round UDC cooling. This maintains the PUE but may reduce the functional duration. Hence, the functional duration i.e. the underwater data center functional duration is considered.

Let ${D}_{1}\left({\alpha }_{n}\right), {\alpha }_{n} \epsilon \alpha$ ${D}_{3}\left({\alpha }_{n}\right)$ denote the expected functional duration for the ${n}{\text{th}}$ underwater data center in phase 1 (NOPh) and phase 3 (ROPh), respectively. The ocean region around the underwater data center ${\mathrm{\alpha }}_{\mathrm{n}}$ experiences marine heat wave events in Phase 2 (MWPh) of its operation. Phase 2 comprises multiple sub-epochs within an epoch. The duration of the marine heat wave event occurring in phase 2 (MWPh) for the underwater data center ${\alpha }_{n}$ between the epochs ${t}_{p}, {t}_{p} \epsilon t$ and ${t}_{x}, {t}_{x} \epsilon t$ is denoted ${D}_{2}\left({\alpha }_{n},{t}_{p},{t}_{x}\right)$. In addition, let ${I}_{re}\left({\alpha }_{n}\right) \epsilon \{\mathrm{0,1}\}$ denote the reservoir status aboard the underwater data center ${\alpha }_{n}$. The underwater data center, ${\mathrm{\alpha }}_{\mathrm{n}}$ does not [existing mechanism without phase 3 (ROPh)] and does use reservoirs [proposed mechanism with Phase 1 (NOPh), Phase 2 (MWPh) and Phase 3 (ROPh)] when ${I}_{re}\left({\alpha }_{n}\right)=0$ and ${I}_{re}\left({\alpha }_{n}\right)=1$, respectively.

The UDC functional duration considering the influence of marine heat waves on the underwater data center in the case of existing mechanism [without phase 3 (ROPh)] and case of proposed mechanism [with phase 3 (ROPh)] are denoted ${\acute{\Gamma}}_{1}$ and ${\acute{\Gamma}}_{1}^{{^{\prime}}}$ respectively:

8 $${{\acute{\Gamma}}}_{1}= \sum_{n=1}^{N}\sum_{k=1}^{Y}\left({D}_{1}\left({\alpha }_{n}\right)+{D}_{3}\left({\alpha }_{n}\right)\right)+ \left({D}_{2}\left({\alpha }_{n},{t}_{k},{t}_{k+j}\right){I}_{re}\left({\alpha }_{n}\right)=0\right), {t}_{k}\epsilon t, {t}_{k+j} \epsilon t , j\ge 1$$

9 $${{\acute{\Gamma}}}_{1}^{^{\prime}}= \sum_{n=1}^{N}\sum_{k=1}^{Y}\left({D}_{1}\left({\alpha }_{n}\right)+{D}_{3}\left({\alpha }_{n}\right)\right)+ \left({D}_{2}\left({\alpha }_{n},{t}_{k},{t}_{k+j}\right){I}_{re}\left({\alpha }_{n}\right)=1\right), {t}_{k}\epsilon t, {t}_{k+j} \epsilon t , j\ge 1$$

Let ${\zeta}_{re}({\alpha }_{n})$ denote the operational efficiency of the reservoir system aboard the underwater data center ${\alpha }_{n}$. An ideal efficiency i.e. ${\zeta}_{re}({\alpha }_{n})=1$ implies that the reservoir system cools the computing payload aboard the underwater data center during the entire duration of the marine heat wave event. A non-ideal efficiency i.e. ${\zeta}_{re}({\alpha }_{n})<1$ implies that the proposed reservoir system is unable to provide water to enable the cooling of the computing payload aboard the underwater date center, ${\alpha }_{n}$ during the entire duration of the marine heat wave event. A non-ideal efficiency may arise when the reservoir capacity is unable to store cold water for server cooling during the duration of the marine heat wave event. The UDC functional duration considering the reservoir system operational efficiency, ${\acute{\Gamma}}_{2}$ is given as:

10 $${\acute{\Gamma}}_{2}= \sum_{n=1}^{N}\sum_{k=1}^{Y}\left({D}_{1}\left({\alpha }_{n}\right)+{D}_{3}\left({\alpha }_{n}\right)\right)+ \left({D}_{2}\left({\alpha }_{n},{t}_{k},{t}_{k+j}\right)\left({I}_{re}\left({\alpha }_{n}\right)=1\right){\mathrm{\zeta}}_{\mathrm{re}}({\mathrm{\alpha }}_{\mathrm{n}})\right), {t}_{k}\epsilon t, {t}_{k+j} \epsilon t , j\ge 1$$

## Performance evaluation and benefit

The performance evaluation is presented in this section. The performance metric is the underwater data center functional duration. This is because the occurrence of marine heat waves in the absence of the proposed reservoir system reduces data center functional duration by making free coolant (freely available ocean cold water) non-available. The non-availability of free coolant causes a non-functioning of the UDC cooling system. The evaluation is done to investigate the UDC functional duration considering that the occurrence of marine heat wave reduces coolant availability and directly influences the functional duration.

The evaluation is done to investigate UDC functional duration via MATLAB stochastic simulation. In the simulation, an underwater data center operational duration of 105 days is considered. This is because of the observation in^13^ that the existing work i.e., Project Natick was functional for 105 days. In addition, the discussion in^15^ notes that marine heat waves can observe for a range of 5–10 days. Furthermore, a maximum duration of marine heat waves of up to 170 days has been observed in^15^. The number of days for which the marine heat wave occurs has been observed to have a minimum of 5 days and a maximum of 170 days as seen in^15^. The choice of the marine heat wave duration has been made considering these occurrence parameters as presented in^15^. Furthermore, the characterization of the types of marine heat waves is a subject that is beyond the scope of the presented research. In addition, the number of day span for marine heat wave occurrence has been less than half of the maximum observed in^13^ of 105 days. This is done to avoid a greedy estimate of the performance benefit of the proposed use of UDC cooling via reservoir systems. In a similar manner, a non-ideal reservoir with efficiencies less than 100% has been considered. Hence, the reservoir efficiency has not consistently and continuously had a value of 100% in the simulation.

This functional duration is divided into equal lengths in Phases 1, 2 and 3. Phases 1 and 3 each have a length of 35 days. The incidence of marine heat waves is considered to last a minimum of 5 days as seen in^41^. The duration of the marine heat waves is deemed the duration of Phase 2. However, the duration of Phase 2 does not exceed 35 days. This consideration has been made to ensure that the total operational duration of 105 days is not exceeded. The evaluation investigates the performance benefit of UDC reservoirs in its influence on the functional duration.

Existing work does not consider the occurrence of marine heat waves and their influence on the functioning of UDCs. The UDC is operational in a variable number of phases each having an equal duration. The phases during which marine heat waves occur have a variable duration and are also described by a given number of epochs.

The simulation is done for the case of a marine heat wave (MHW) event comprising ten epochs and fifteen epochs in the simulation procedure. In each epoch, the underwater data center functions in a varying number of phases i.e. 3 phases, 4 phases, 5 phases and 6 phases. In the case of 3 phases, 4 phases, 5 phases, and 6 phases, the duration of each non-MHW occurring phase are 35 days, 26.25 days, 21 days and 17.5 days, respectively. The duration of the MHW occurring phase exceeds the non-MHW occurring phase duration. The occurrence of MHW is limited to a phase out of the total number of phases with epochs using the parameters in Table 1.

**Table 1:** System simulation parameters.

Parameter	3	4	5	6
**Number of MHW occurring phase. Each phase has 10 epochs**				
Maximum MHW duration (days)	33.7	25.3	20.2	16.9
Minimum MHW duration (days)	7.7	5.8	4.6	3.9
Mean MHW duration (days)	19.2	14.4	11.5	9.6
Maximum reservoir efficiency (%)	91.7	91.7	91.7	91.7
Minimum reservoir efficiency (%)	16.2	16.2	16.2	16.2
Mean reservoir efficiency (%)	53.6	53.6	53.6	53.6
**Number of MHW phases. Each phase has 15 epochs**				
Maximum MHW duration (days)	34.9	26.2	20.9	17.5
Minimum MHW duration (days)	0.8	0.6	0.5	0.4
Mean MHW duration (days)	17.9	13.4	10.8	9.0
Maximum reservoir efficiency (%)	97.9	97.9	97.9	97.9
Minimum reservoir efficiency (%)	4.3	4.3	4.3	4.3
Mean reservoir efficiency (%)	55.3	55.3	55.3	55.3

**Figure 9:**
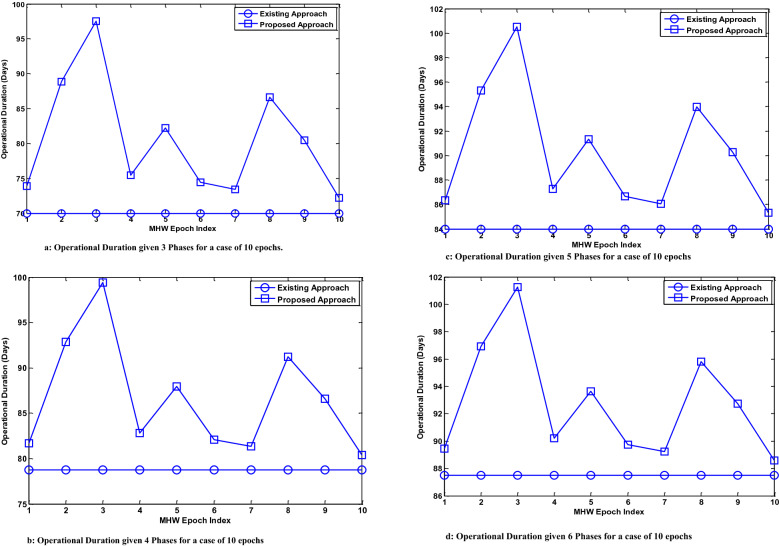
(**a**) Operational Duration given 3 Phases for a case of 10 epochs. (**b**) Operational Duration given 4 Phases for a case of 10 epochs. (**c**) Operational Duration given 5 Phases for a case of 10 epochs. (**d**) Operational Duration given 6 Phases for a case of 10 epochs. Evaluation showing results for the underwater data center duration (for 3 phases, 4 phases, 5 phases and 6 phases) in the case that the marine heat wave event occurs for 10 epochs.

**Figure 10:**
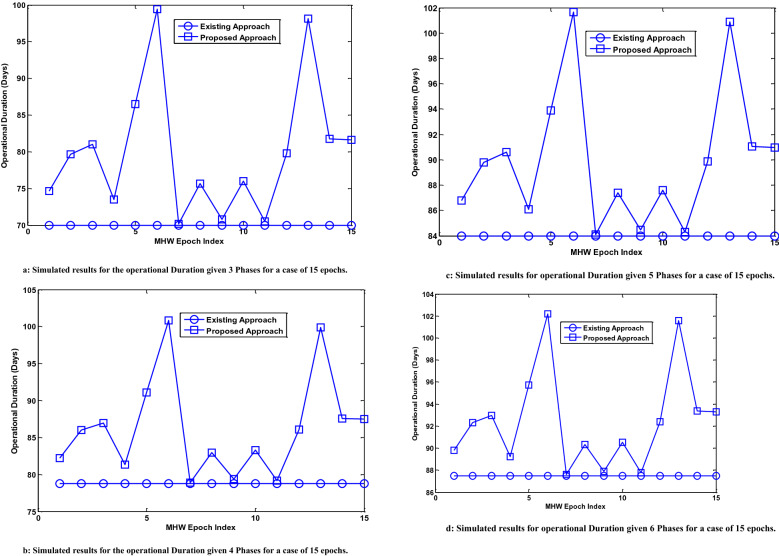
(**a**) Simulated results for the operational Duration given 3 Phases for a case of 15 epochs. (**b**) Simulated results for the operational Duration given 4 Phases for a case of 15 epochs. (**c**) Simulated results for operational Duration given 5 Phases for a case of 15 epochs. (**d**) Simulated results for operational Duration given 6 Phases for a case of 15 epochs. Evaluation showing results for the underwater data center duration (for 3 phases, 4 phases, 5 phases and 6 phases) in the case that the marine heat wave event occurs for 15 epochs.

The operational duration when the MHW phase comprises 10 epochs and 15 epochs are presented in Figs. 9 and 10, respectively. The simulation results that have been presented in Figs. 9 and 10 describe how the UDC operational duration is influenced by the occurrence of marine heat waves in the underwater environment. In all the presented results, the operational duration is examined in the case of the existing approach (without the use of proposed reservoirs) and proposed approach (with the use of proposed reservoirs). The performance simulation result for the operational duration in the case of the existing mechanism is observed to be constant. This is because of the non-consideration of the dynamic reservoir efficiency in the case of existing mechanism. In the presented results, the case of a UDC operational in the underwater environment considering the occurrence of marine heat waves in the absence of the proposed solution is described as the Existing Approach. The results being presented in the case of the Proposed Approach describe the UDC operational duration considering the occurrence of marine heat waves when the proposed solution is incorporated.

From the results, it can be seen that the total duration in all cases is less than 105 days. This is due to the occurrence of the MHW event. Figure 9 has four sub-figures i.e. Fig. 9a–d. The simulation results obtained given 10 epochs for the case of 3 phases, 4 phases, 5 phases and 6 phases are presented in Fig. 9a–d, respectively. Figure 10 also has four sub-figures i.e. Fig. 10a–d. The simulation results presented in Fig. 10 consider the case where the MHW occurring phase has 15 epochs. The results obtained given 15 epochs for the case of 3 phases, 4 phases, 5 phases and 6 phases are presented in Fig. 10a–d, respectively.

The performance improvement i.e. the mean increase in the operational duration of the underwater data center is also analyzed using the simulation results. This is done for the case of 10 epochs (in non-MHW occurring phase) and 15 epochs (in non-MHW occurring phase). Given that the MHW occurring phase has 10 epochs, the use of the proposed solution increases the underwater data center operational duration by 12.3%, 8.7%, 6.7% and 5.5% for the case of 3 phases, 4 phases, 5 phases and 6 phases, respectively.

The use of the proposed solution results in the highest improvement in the operational duration with the least number of operational phases. The highest improvement in the UDC operation is obtained with the least number of phases. In the case of the least number of phases, the MHW occurring phase has the longest expected operation. This implies that the use of the reservoirs as proposed enables the cooling of the underwater data centers for the longest duration in the case where there is a fewer number of operational phases.

Analysis of the results presented in Fig. 10a–d shows the obtained operational duration when an MHW occurring phase has 15 epochs. In a similar manner, the operational duration of the underwater data center is less than 105 days. This is due to the consideration of the occurrence of MHW in the simulation. The mean percentage improvement in the underwater data center operational duration due to the use of the proposed solution is also investigated. The use of the proposed solution increases the operational duration by 11.5%, 8.2%, 6.3% and 5.2% on average for the case of 3 phases, 4 phases, 5 phases and 6 phases, respectively.

## Conclusion

The research being presented proposes a solution enabling the functioning of underwater data centers when marine heat waves occur. Marine heat waves affects all ocean regions especially those closer to the surface i.e. the epipelagic and mesopelagic ocean zones. Underwater data centers that are placed in the epipelagic and mesopelagic zones experience an insufficient supply of cold water required for cooling due to the occurrence of marine heat waves. The reduced cooling capability can potentially limit the operational duration of underwater data centers. The solution being proposed incorporates temperature sensors enabling the detection of the occurrence of anomalous temperature increases in the ocean region hosting underwater data center systems. In addition, the proposed underwater data center also incorporates reservoirs that store cold water. The stored cold water is used to cool underwater data centers and ensure their continued operation when marine heat waves occur. The use of the proposed solution increases the underwater data center operational duration. The operational duration of the underwater data center considering the occurrence of marine heat waves in a given phase comprising multiple epochs is investigated via simulation. The operational duration is enhanced by at least 5.2% and at most by 12.3% on average by using the proposed solution.

## Data Availability

All data generated or analysed during this study are included in this published article [and its supplementary information files].
